# Feasibility, safety, and long-term efficacy of stereotactic radiofrequency ablation for tumors adjacent to the diaphragm in the hepatic dome: a case-control study

**DOI:** 10.1007/s00330-019-06399-y

**Published:** 2019-09-05

**Authors:** Peter Schullian, Daniel Putzer, Gregor Laimer, Elliot Levy, Reto Bale

**Affiliations:** 1 Department of Radiology, Section of Interventional Oncology - Microinvasive Therapy, Medical University of Innsbruck, Anichstr. 35, 6020 Innsbruck, Austria; 2 Radiology and Imaging Sciences, National Institutes of Health, Bethesda, MD USA

**Keywords:** Radiofrequency ablation, Liver, Neoplasms

## Abstract

**Objectives:**

Achievement of adequate treatment margins may be challenging when the target is either difficult to visualize, awkward to access, or situated adjacent to vulnerable structures. Treatment of tumors located close to the diaphragm in the hepatic dome is challenging for percutaneous radiofrequency (RF) ablation for these reasons. The purpose was to assess the feasibility, safety, and clinical outcome of multi-probe stereotactic RF ablation (SRFA) of liver tumors in the subdiaphragmatic area.

**Methods:**

Between 2006 and 2018, 177 patients (82 HCCs, 6 ICCs, and 89 metastatic tumors) underwent SRFA of 238 tumors abutting the diaphragm in the hepatic dome. For comparison, 177 patients were randomly selected from our database by the R package “MatchIt” for propensity score matching to compare treatment safety and efficacy in this retrospective, single-center study.

**Results:**

Median treated tumor size was 2.2 cm (range 0.5 to 10 cm). SRFA was primarily successful for 232/238 (97.5%) tumors. Five tumors were successfully retreated, resulting in a secondary technical efficacy rate of 99.6%. Local tumor recurrence developed in 21 of 238 tumors (8.8%). The major ablation complication rate was 10.7% (22 of 204). Twelve (55%) of 22 major complications could be successfully treated by the interventional radiologist in the same anesthesia session. There was no significant difference in adverse events or disease control rates between the subdiaphragmatic tumors and matched controls.

**Conclusions:**

SRFA is a safe and feasible option in the management of difficult-to-treat tumors abutting the diaphragm in the hepatic dome, with similar safety profile compared with matched controls.

**Key Points:**

*• RFA was primarily successful for 232/238 (97.5%) subdiaphragmatic dome tumors. Local tumor recurrence developed in 21 of 238 tumors (8.8%).*

*• The major complication rate directly related to ablation of the hepatic dome tumors was 10.7% (22 of 204). 12/22 (55%) of major complications could be successfully treated in the same anesthesia session.*

*• There was no significant difference in adverse events or disease control rates between the subdiaphragmatic tumors and matched controls.*

## Introduction

RF ablation has been increasingly accepted as a curative alternative to surgical resection in the management of primary or metastatic liver tumors [1, 2]. The achievement of a sufficient safety margin, i.e., complete coverage of the coagulation zone with a margin of at least 5 mm, is crucial for good local tumor control and clinical outcome [3]. Treatment of subdiaphragmatically located tumors located in the hepatic dome is challenging when the target tumor is difficult to visualize, awkward to access, or situated adjacent to vulnerable structures. Treatment of tumors located close to the diaphragm implies a higher risk of complications such as pneumothorax or diaphragmatic injuries. Several studies reported severe right shoulder pain after treatment of hepatic dome tumors [4, 5].

Common strategies include angulated approach sparing the pleura [5] or transthoracic or transpleural access [6–8]. Hydrodissection is another possible protective method in the hepatic dome [9]. In addition, these tumors located posteriorly and superiorly also pose a significant technical challenge for laparoscopic liver resection, demanding special approaches and techniques [10]. There are several studies reporting limited local tumor control in subcapsular locations with conventional RF ablation [11], higher complication rates (mainly bleeding), as well as tumor recurrence [12, 13]. Stereotaxy has proven useful for planning and execution of complex or difficult ablations. Difficult access routes may be specifically facilitated and more precise coverage of the target tumor and safety margin accomplished with frameless stereotactic navigation systems [14].

The purpose of the current study was to assess the feasibility, safety, and clinical outcome of multi-probe SRFA of liver tumors close to the diaphragm and to compare the results with a matched control group. Our hypothesis was that there is no difference in terms of safety and local control for tumors in the hepatic dome vs. non-hepatic dome tumors.

## Materials and methods

### Patient cohort and inclusion criteria

**Fig. 1:**
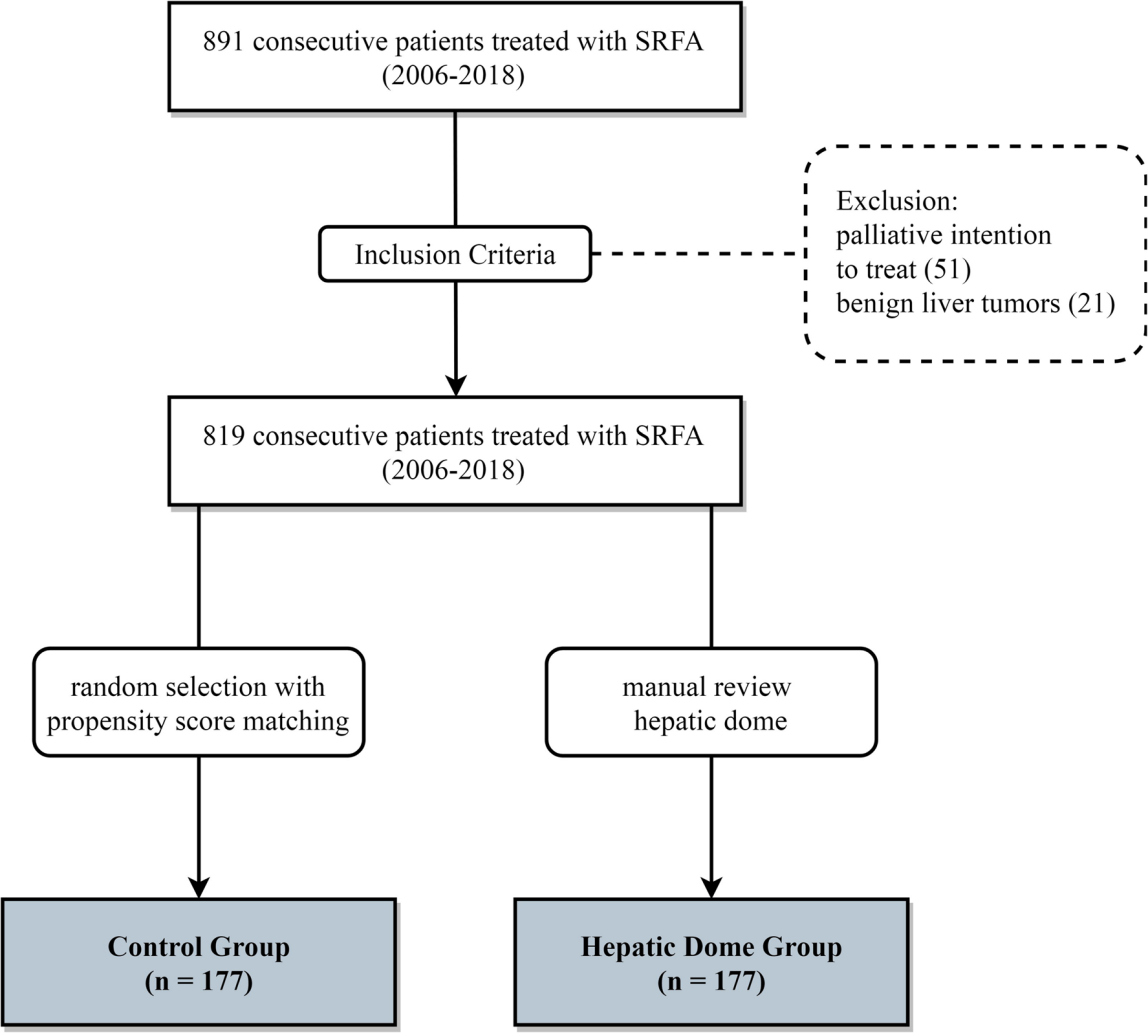
Flowchart of group assignment

This retrospective, single-center study was approved by the Institutional Review Board. Written informed consent for SFRA was obtained from all patients. All cases were reviewed and treatment plans approved by consensus in multi-disciplinary tumor board meetings. Between 2006 and 2018, 891 consecutive patients were treated by SRFA. Seventy-two patients with invasion of the portal vein, extended tumor spread with subsequent palliative intention to treat, or benign liver tumors were excluded (Fig. 1). A total of 177/819 patients with hepatic dome tumors were included. A total of 177 hepatic ablation patients were selected using nearest neighbor propensity score matching by the R package “MatchIt” with sex, age, tumor type, number and size, and liver function as matching variables. The baseline characteristics of the two groups are shown in Table 1.

**Table 1:** Patient characteristics of 177 patients undergoing 204 SRFA sessions of 238 nodules in the hepatic dome group and of 177 patients undergoing 281 SRFA sessions of 587 nodules in the control group

Patient characteristics	Hepatic dome gr.	Control gr.
Age, median years (range)	65 (7–87)	64 (25–82)
Sex (female/male), *n* (%)	48/129 (27.1/72.9)	48/129 (27.1/72.9)
Tumor type, *n* (%)		
- HCC, *n* (%) - ICC, *n* (%) - Metastasis, *n* (%) • Colorectal, *n* (%) • Breast, *n* (%) • Melanoma, *n* (%) • Other, *n* (%)	82 (46.3) 6 (3.4) 89 (50.3) 52 (65.8) 5 (6.3) 7 (8.9) 25 (19)	80 (45.2) 11 (6.2) 86 (48.6) 47 (61.8) 3 (3.9) 4 (5.3) 32 (29)
Cirrhosis, *n* (%) - Child A, *n* (%) - Child B, *n* (%) - Child C, *n* (%)	68 (38.4) 60 (88.0) 7 (11.1) 1 (0.9)	66 (37.3) 54 (81.1) 11 (16.8) 1 (2.1)
Tumor size, median (range)	2.2 cm (0.5–10 cm)	2.0 cm (0.5–13 cm)
Tumor number at beginning, *n* (range)	2 (1–8)	2 (1–9)
Total treated tumors, median (range)	1 (1–4)	2 (1–20)
Ablations per patient, *n* (range)	1 (1–4)	1 (1–10)
Patients receiving LTX, *n* (%)	23 (13.1)	24 (13.6)

*SRFA* stereotactic radiofrequency ablation, *HCC* hepatocellular carcinoma, *ICC* intrahepatic cholangiocarcinoma, *Child* Child-Pugh score, *LTX* liver transplantation, *gr.* group

Exclusion criteria for SRFA were a platelet count of < 50,000/mm^3^ and prothrombin activity < 50%. Tumor diagnosis was confirmed by classic tumor enhancement pattern on multi-phasic contrast MRI or CT and pathologically validated by needle biopsy during the RF ablation. The hepatic dome was defined as the portion of the liver located close to the diaphragm, accounting for almost one-third of the total liver volume.

### Multi-probe stereotactic radiofrequency ablation

The method of SRFA has been described previously [15, 16]. Patients under general anesthesia were immobilized on the CT table by a single- (Bluebag, Medical Intelligence) or double-vacuum fixation technique (BodyFix, Medical Intelligence) with paralysis. For image registration, 10–15 optical fiducials, (X-SPOT, Beekley Corporation) were broadly attached to the skin of the thorax and upper abdomen.

**Fig. 2:**
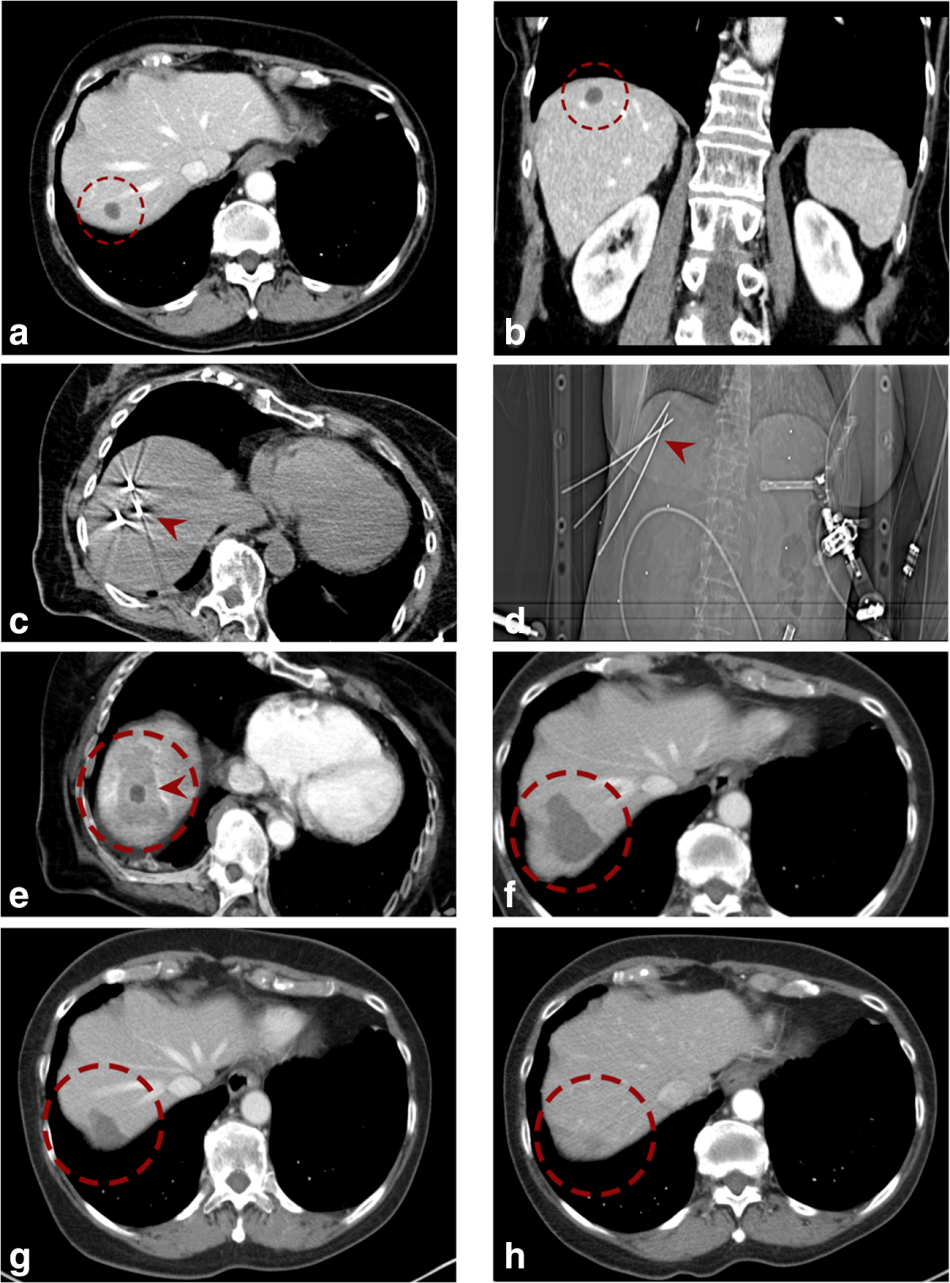
Case of a 70-year-old female with a 1.5-cm colorectal cancer liver metastasis in segment VII in the hepatic dome. **a**, **b** Portal venous phase initial CT scans with a hypo-enhancing nodule in segment VII (*red dashed circle*). **c**, **d** Axial native control CT and scout (**d**) with 3 coaxial needles in place (*red arrowhead*). **e** Fused CT image of the contrast-enhanced planning and final control CTs showing a complete coverage of the tumor (*dark central nodule*) by the coagulation zone (*red arrowhead*). **f**–**h** The *red dashed circle* is marking the progressively shrinking coagulation zone at 3 months (**f**), 24 months (**g**), and 48 months (**h**) after SRFA

**Fig. 3:**
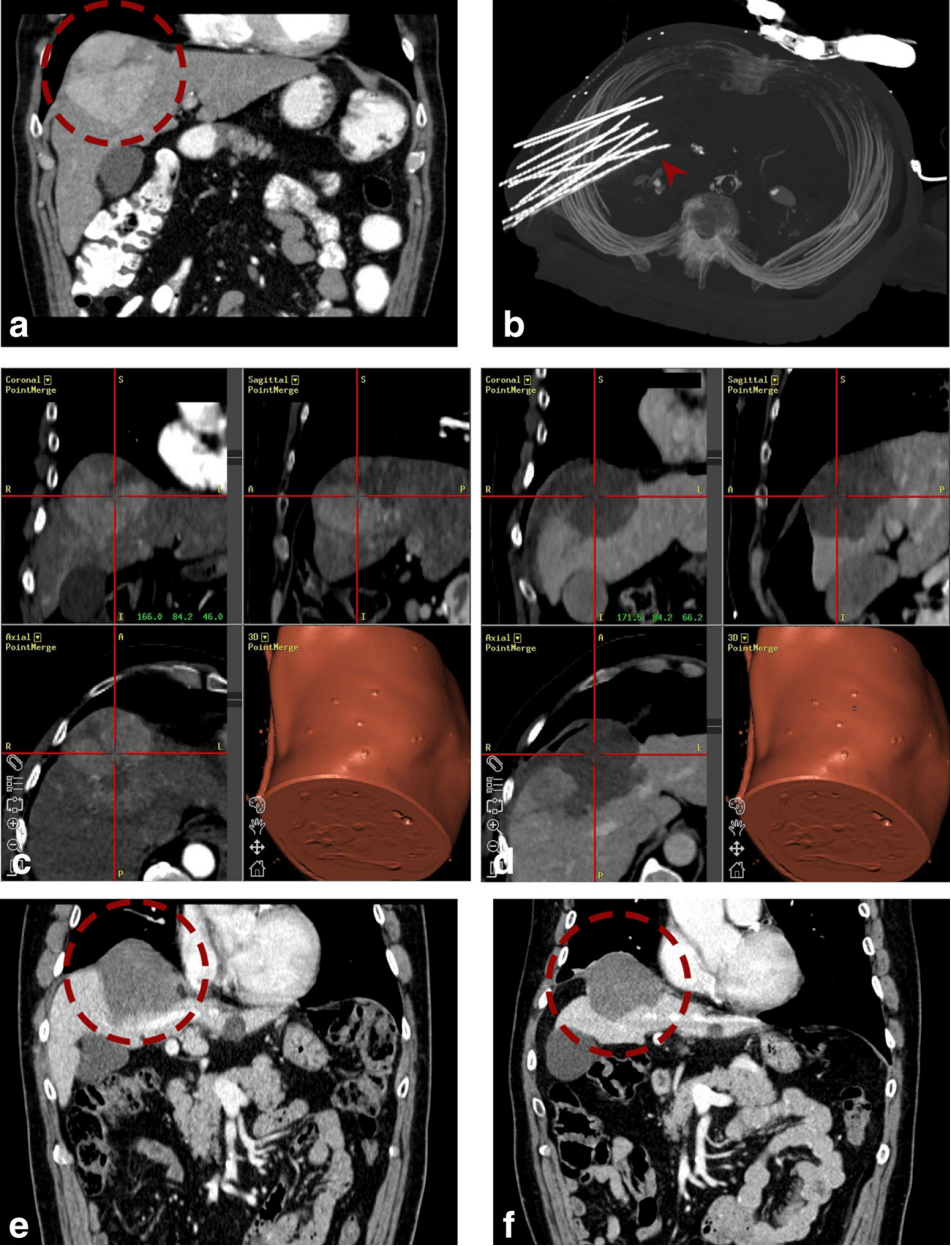
Case of a 67-year-old male with a large HCC in the hepatic dome. **a** Arterial phase planning CT with a 9-cm HCC in segment IV/VIII (*red dashed circle*). **b** MIP of the control CT showing in total 12 inserted coaxial needles (*red arrowhead*). **c**, **d** Fused images from the navigation system with 3D views from arterial phase planning CT (**a**) and final control CT (**d**) with complete necrosis including a sufficient ablation margin. **e**, **f** Follow-up CT scans after 3 (**e**) and 24 months (**f**) with no evidence of local tumor recurrence (*red dashed circle* is marking the coagulation zone)

A contrast-enhanced planning CT (Siemens SOMATOM Sensation Open, sliding gantry with 82-cm diameter, Siemens AG) was obtained with 3-mm slice thickness in exhalatory phase. To improve image registration, the endotracheal tube (ETT) was transiently disconnected during the planning CT, each stereotactic needle placement, and the final control CT. The pretreatment CT datasets were transferred to the optical-based navigation system (Stealth Station Treon Plus, Medtronic Inc.). All nodules abutting the diaphragm in the hepatic dome were targeted with an angulated either sub- or intercostal approach avoiding the pleural recess. SRFA probe trajectories were planned using multi-planar and 3D reconstructed images. After automatic registration and a registration accuracy check, 15-G/17.2-cm coaxial needles (Bard Inc.) were advanced through an ATLAS aiming device (Medical Intelligence Inc.) without real-time imaging control. The ATLAS aiming device consists of two joints and a bracket holder with an adjustable concentric aperture for the use of different instruments. It is mounted to a mechanical arm with three joints and six degrees of freedom allowing for precise trajectory alignment. The depth from the aiming device to the target was automatically calculated by the navigation software. In order to achieve complete necrosis of the entire tumor tissue with an appropriate circumferential safety margin, RFA electrodes were aligned to each other with a maximum separation distance of 2 cm. For verification of correct needle placement, a native control CT was performed and fused with the planning CT using the navigation system’s image 3D registration algorithm. A 16-G coaxial biopsy sample was obtained in all cases. 17-G RF electrodes (Cool-tip, Medtronic, 25-cm length and 3-cm exposure) were then introduced through the coaxial needles for serial tumor ablation. RF ablation was carried out using the unipolar Cool-tip_RF generator (Cool-tip, Medtronic), including the Cool-tip_RF switching controller. The ablation time for three electrodes (switching control) was 16 min. Finally, an immediate contrast-enhanced CT scan was performed and fused with the planning CT for verification of the ablation size and to exclude possible complications. Needle track ablation was done during repositioning and during final removal of the RF electrodes to prevent bleeding and potential tumor seeding. Example images from multi-probe SRFA in the hepatic dome are shown in Figs. 2 and 3.

The study endpoints of primary and secondary technical efficacy and local recurrence rate (LR) were determined by follow-up contrast-enhanced CT or MR scans performed at 1-month and at 3-month intervals after RF ablation. The imaging results were evaluated by two abdominal radiologists with more than 10 years of experience by consensus (PES and BR). Technical success was defined as sufficiently accurate (deviation < 1 cm at the needle tip) electrode placement according to predefined plans. Complete ablation was defined as a circumscribed non-enhancing zone within and/or extending beyond the initial tumor borders with a well-defined margin. Appearance of new nodules within or directly adjacent to the ablation zone or to initial tumor was judged to constitute LR. New nodules distant to the ablation zone and/or to initial tumor location were defined as distant tumor recurrence. Primary technical efficacy rate was evaluated per tumor as the absence of residual tumor on 1-month follow-up CT. Secondary technical efficacy rate included tumors that demanded re-ablation due to residual tumor. Complications were defined according to the Society of Interventional Radiology (SIR) Standards of Practice Committee classification [17]. The secondary study endpoints included disease-free (DFS) and overall survival (OS). In patients with exclusively hepatic dome HCC tumors and accordingly matched controls, survival was calculated from the date of initial stereotactic RF ablation to the date of death attributable to malignancy or other causes (i.e., event) or to the most recent follow-up visit (i.e., censoring).

### Statistical analysis

The software R (version 3.5.2, R Foundation for Statistical Computing) and the R package MatchIt (1:1 matching with the nearest neighbor) were used for the propensity matching process to select patients for the control group.

The statistical analysis of comparisons was performed with the software IBM SPSS version 20 (IBM). Data were expressed as total numbers, median, and range. The overall survival and disease-free survival were evaluated using the Kaplan-Meier method and compared between the two groups with the log-rank test. The difference between categorical variables was evaluated with the *X*^2^ test, and the difference between independent continuous variables was evaluated with the Mann-Whitney *U* test. A *p* value < 0.05 was considered as with statistical significance.

## Results

### Patient characteristics (Table 1)

A total of 177 patients, 48 females and 129 males, with a median age of 65 years (7–87) underwent SRFA for treatment of subphrenic tumors in the hepatic dome. Diagnoses included 82 (46.3%) HCCs, 6 (3.4%) ICCs, and 89 (50.3%) metastatic tumors. The majority (65.8%) of metastatic disease originated from colorectal cancer. The median size of the 238 nodules was 2.2 cm (0.5–10 cm). A median of 2 tumors (1–11) were treated per ablation session (in total 204 sessions), including 111 patients (62.7%) with additional tumors outside of the hepatic dome. At the beginning of the treatment, 76 patients had a solitary tumor in the liver, 49 had two tumors, 24 had three tumors, and 28 had more than three tumors (multiple nodules). Sixty-eight (38.4%) patients suffered from underlying liver cirrhosis (60 (88%) Child-Pugh A), and 23 (13.1%) received a liver transplantation during follow-up. Forty-one patients underwent chemotherapy, 25 patients surgical resection, 9 patients TACE, and 6 patients conventional RF ablation prior to SRFA.

### Perioperative complications

Perioperative major complications are shown in detail in Table 2. One death occurred following ablation of colorectal liver metastasis due to major bleeding (mortality rate 0.5% (1/204)). The total major complication rate was 12.3% (25 of 204). Three of these complications were clearly related to simultaneous thermal ablation of tumors in other locations, leading to a major complication rate of 10.7% (22 of 204) in hepatic dome tumors.

**Table 2:** Details of major complications after SRFA

Patient	Age	Sex	Primary tumor	Cirr.	Tumors/session	Lesion size (max/session)	Needles/session	Complication	Therapy	Related to H.D.
1	53	Male	HCC	Yes	2	5.0 cm, (5.0 cm)	16	Liver failure	Acute LTX	Yes
2	48	Male	HCC	No	1	6.4 cm, (6.4)	12	ARDS, pleural effusion	ICU, drainage	Yes
3	73	Female	HCC	Yes	1	1.1 cm	3	Diaphragmatic defect	Surgery	Yes
4	47	Male	NET	No	2	3.5 cm, (3.5)	6	Diaphragmatic hernia	Surgery	Yes
5	68	Female	OVC	No	2	2.8 cm, (2.8)	6	Bowel thermal damage	Surgery	No
6	77	Male	CRC	No	3	5.5 cm, (5.5)	12	Perihepatic bleeding, hemorrhagic shock w. death	AG-coiling, ICU	Yes
7	69	Male	CRC	No	1	7.2 cm	10	Pneumothorax	Chest-tube	Yes
8	61	Male	CRC	No	2	4.0 cm, (4.0)	8	Pneumothorax	Chest-tube	Yes
9	61	Female	CRC	No	5	5.0 cm, (5.0)	22	Pneumothorax	Chest-tube	Yes
10	70	Male	HCC	Yes	1	7.0 cm	9	Pneumothorax	Chest-tube	Yes
11	69	Male	HCC	Yes	3	4.0 cm, (4.0)	14	Pneumothorax	Chest-tube	Yes
12	38	Male	CRC	No	5	1.0 cm	14	Perihepatic bleeding	AG-coiling	No
13	72	Male	ESC	No	1	1.5 cm	5	Perihepatic bleeding	AG-coiling	Yes
14	51	Male	HCC	Yes	1	1.5 cm	3	Perihepatic bleeding	AG-coiling	Yes
15	72	Male	CRC	No	4	2.0 cm, (2.1)	15	Perihepatic bleeding	AG-coiling	Yes
16	46	Female	OVC	No	2	3.0, (3.0)	7	Perihepatic bleeding	AG-coiling	Yes
17	63	Male	HCC	Yes	2	2.0 cm, (2.7)	4	Intrahepatic bleeding	AG-coiling	Yes
18	63	Male	HCC	Yes	1	2.2 cm	3	Perihepatic bleeding	AG-coiling	Yes
19	48	Male	HCC	No	3	2.0 cm, (3.0)	9	Perihepatic bleeding	AG-coiling	No
20	7	Female	NBL	No	1	3.5 cm	6	Intrahepatic bleeding	AG-coiling	Yes
21	67	Male	HCC	Yes	2	1.6 cm, (1,8 cm)	6	Pleural effusion	ICU, drainage	Yes
22	56	Male	HCC	Yes	3	2.8 cm (2.8 cm)	10	Pleural effusion	Drainage (US-guided)	Yes
23	60	Male	CRC	No	1	3.0 cm	10	Pleural effusion	Drainage (US-guided)	Yes
24	68	Male	MEL	No	1	5.5 cm	8	Pleural effusion	Drainage (US-guided)	Yes
25	58	Male	HCC	Yes	2	8.5 cm, (8.5)	18	Pleural effusion	Drainage (US-guided)	Yes

*SRFA* stereotactic radiofrequency ablation, *HCC* hepatocellular carcinoma, *CRC* colorectal carcinoma, *MEL* melanoma, *NBL* neuroblastoma, *NET* neuroendrocrine tumor, *OVC* ovarian cancer, *ESC* esophageal cancer, *Cirr* hepatic cirrhosis, *ICU* intensive care unit, *AG* angiography, *LTX* liver transplantation, *H.D.* hepatic dome

Thermal injuries of the diaphragm lead to a local defect in 2 cases that had to be surgically repaired. One patient developed liver failure after treatment of 2 HCCs (5 cm and 3 cm) requiring salvage liver transplantation. In one case, thermal injury of the bowel had to be surgically repaired. However, this complication was related to simultaneous thermal ablation of an additional tumor in segment VI in the same session. Other complications included transient pulmonary failure with bilateral effusions (1 patient) pleural effusions requiring thoracenteses (5 patients).

A total of 14/25 (56%) major complications were successfully treated by the interventional radiologist in the same anesthesia session by placing a thoracostomy tube in 5 patients with pneumothoraces and by transarterial embolization in nine patients with hepatic hemorrhages, respectively. Fever (> 37 °C) and variably pronounced right shoulder pain (mainly mild) developed in all patients but subsided within a few days with symptomatic treatment. This is most likely related to thermal injury of the adjacent diaphragm and pleura. The median hospital stay after the ablation was 4 days, ranging from 1 to 28 days. There was no significant difference of major complication rate (8.5%, 24/281; *p* = 0.180) and hospital stay (median 4, 1–42 days; *p* = 0.301) compared with the control group, respectively.

A subanalysis of the major complications in the hepatic dome group showed that the median number of applied RF probes and the median tumor size were significantly higher in ablation sessions with major complications (median 5 vs. 3 RF probes with *p* = 0.034 and median size 3 cm vs. 2 cm with 0.023, respectively).

### Technical success

SRFA was successfully completed according to plan in all 238 tumors (technical success rate 100%). A total of 232/238 tumors were successfully ablated at initial SRFA (97.5% primary technical efficacy rate). Five tumors required retreatment, resulting in a secondary technical efficacy rate of 99.6% (Table 3). One to 20 (median 3) RF electrodes were inserted in each tumor.

**Table 3:** Tumor-based therapy success rates compared with control group

Rate	Hepatic dome gr.	Control gr.	*p* value
Technical success, *n* (%)	238/238 (100)	587/587 (100)	N/A
Primary technical efficacy, *n* (%)	232/238 (97.5)	568/587 (96.8)	0.608
Secondary technical efficacy, *n* (%)	237/238 (99.6)	577/587 (98.3)	0.150
Local recurrence, *n* (%)	21/238 (8.8)	42/587 (7.2)	0.414

*gr.* group

Compared with the control group, there was no significant difference between primary and secondary efficacy (96.8%, 568/587, *p* = 0.608; 98.3%, 577/587, *p* = 0.150).

In addition to SRFA, 3 patients received TACE and 8 patients received chemotherapy. Liver transplantation was performed in 23 patients where the histopathology examination revealed 2 LR.

### Local recurrence rate and distant recurrence

Local tumor recurrence developed in 21 of 238 tumors (8.8%, Table 4, median imaging follow-up 12.6 months). Distant tumor recurrence in the liver was found in 84 patients (47.5%), and extrahepatic metastasis in 23 patients (13%), respectively. Of the 101 patients (57%) experiencing intrahepatic recurrences, including four patients with local and distant recurrences, 82 (81%) patients received repeated SRFA. Thirty-four (41%) patients developed untreatable tumor progression.

**Table 4:** Unsuccessful local tumor control after SRFA

Patient	Age	Sex	Tumor	Cirr.	Tumor size	*N* needles	Ablation time	Unsuccessful Pretherapy	Outcome
1	65	Male	HCC	Yes	7.0 cm	5	32 min	No	LR
2	74	Male	HCC	No	7.0 cm	8	72 min	No	LR
3	75	Male	ICC	No	6.0 cm	5	34 min	No	LR
4	54	Male	CRC	No	10.0 cm	10	112 min	No	LR
5	38	Male	CRC	No	1.0 cm	1	12 min	SRFA	LR
6	57	Male	HCC	Yes	2.6 cm	3	16 min	No	LR
7	69	Male	HCC	Yes	2.0 cm	3	16 min	No	LR
8	69	Male	HCC	Yes	1.0 cm	1	12 min	No	LR
8	69	Male	HCC	Yes	1.0 cm	1	12 min	SRFA	LR
9	60	Male	HCC	Yes	2.8 cm	3	16 min	No	LR
10	66	Female	HCC	Yes	4.5 cm	8	66 min	No	LR
11	48	Male	HCC	No	6.4 cm	12	128 min	No	LR
11	48	Male	HCC	No	5.0 cm	9	64 min	SRFA	LR
12	69	Male	HCC	Yes	5.0 cm	9	96 min	TACE	LR
13	61	Male	CRC	No	4.0 cm	5	29 min	No	LR
13	61	Male	CRC	No	2.5 cm	5	26 min	SRFA	LR
14	68	Male	MEL	No	5.5 cm	8	36 min	No	LR
15	73	Male	HCC	Yes	5.0 cm	9	39 min	No	LR
16	47	Male	NET	No	3.5 cm	3	32 min	No	LR
16	47	Male	NET	No	2.0 cm	2	24 min	No	LR
17	55	Female	OVC	No	2.5 cm	4	32 min	No	LR
18	68	Male	RCC	No	1.0 cm	3	12 min	No	IN
19	65	Male	HCC	Yes	1.3 cm	2	12 min	No	IN
20	49	Male	CRC	No	2.3 cm	2	12 min	No	IN
5	38	Male	CRC	No	2.0 cm	1	12 min	No	IN
21	58	Male	CRC	No	7.0 cm	9	66 min	No	IN
21	58	Male	CRC	No	5.0 cm	7	52 min	No	IN

*SRFA* stereotactic radiofrequency ablation, *HCC* hepatocellular carcinoma, *CRC* colorectal carcinoma, *ICC* intrahepatic cholangiocarcinoma, *MEL* melanoma, *NET* neuroendocrine tumor, *OVC* ovarian cancer, *RCC* renal cell cancer, *Cirr.* hepatic cirrhosis, *IN* incomplete necrosis, *LR* local recurrence, *TACE* transcatheter arterial chemoembolization

The local recurrence rate of the control group was 7.2% (42/587). There was no significant difference compared with the hepatic dome group (*p* = 0.414).

### Overall and disease-free survival (Fig. 4)

In patients with exclusively HCCs in the hepatic dome, the overall survival rates at 1, 3, and 5 years from the date of the first SRFA were 87.1%, 76.2%, and 58.2% and 91%, 75.8%, and 55.2% for matched controls, with a median overall survival (OS) of 66.9 months (95% CI 50.4–84.7) and 72 months (95% CI 36.6–142), respectively. The disease-free survival (DFS) for patients with exclusively HCCs in the hepatic dome after SRFA was 78%, 47.5%, and 35.6%, at 1, 3, and 5 years, respectively, with a median DFS of 19.2 months (95% CI 16.6–52.9) and for matched controls 67.2%, 50.8%, and 35.5%, with a median DFS of 36.6 months (95% CI 12.1–53.8), respectively.

**Fig. 4:**
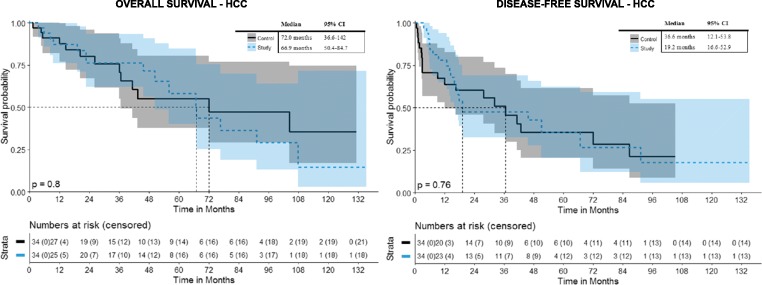
Overall and disease-free survival after initial SRFA of patients with exclusive HCCs in the hepatic dome in comparison with their matched controls

In terms of OS and DFS, there was no significant difference between the control and hepatic dome groups, with *p* = 0.804 and *p* = 0.757, respectively.

## Discussion

This retrospective analysis with a propensity score–matched control cohort suggests that SRFA of subphrenic tumors can be accomplished with comparable safety and efficacy rates achieved for tumors treated elsewhere in the liver. The technical success rate, i.e., completion according to plan, in our study was 100%, which is in line with reports from the literature [5, 18]. The primary technical efficacy was 97.5%, also comparable with reported primary technical efficacy rates after conventional RF ablation in the hepatic dome ranging from 86.7% [5] to 93.2–96% [19, 20]. The conventionally treated tumors were smaller (median diameter 1.7 cm and maximal diameter 4 cm) compared with our study with a median tumor size of 2.2 cm and a maximal diameter of 10 cm. We observed local tumor progression in 21 (8.8%) of 238 tumors, with no significant difference in local tumor treatment outcomes compared with the control group (*p* = 0.414). Kim et al [5] reported local tumor progression in 13.3% after RF ablation of subdiaphragmatic HCCs with a transthoracic approach despite the inclusion of smaller lesions. Cha et al [21] compared the therapeutic outcomes of conventional US-guided RF ablation for subcardiac and non-subcardiac HCCs in 73 patients and reported no significant difference observed between both groups, with a cumulative local tumor progression rate > 15% in the subcardiac group. Recently, Vo Chieu et al [18] showed local tumor progression rates of up to 13.6% in the “risk group” after microwave ablation of tumors abutting the diaphragm. Microwave ablation (MWA) generally requires fewer overlapping ablations compared with RFA. Asvadi et al [22] treated 48 hepatic dome lesions in 46 patients with conventional CT-guided MWA and reported a complete response rate of 94%. Transarterial chemoembolization (TACE) is the recommended locoregional treatment for patients with intermediate HCC [23, 24]. Several studies reported the feasibility and benefit of RF ablation after transarterial embolization with complete response rates of 76–82% in large HCC [25, 26]. We attribute the good local tumor control after SRFA to consistent achievement of a sufficient safety margin of at least 5 mm [3]. SRFA offers three-dimensional ablation planning to achieve optimal alignment of RF probes to create multiple overlapping coagulation volumes. Usage of an aiming device facilitates very precise path alignment and targeting [14]. In case of poor tumor visibility, SRFA planning may include fusion with previously acquired MR images. Temporary disconnection of the endotracheal tube facilitates good control of respiratory motion [27]. Immediate postablation contrast-enhanced CT fusion with the planning CT allows for rapid reliable judgment of the ablation results with the option for re-ablation. Bale et al [28] achieved complete pathological response after SRFA (i.e. no evidence of tumor) with these techniques in 183 of 188 (97.3%) hepatocellular carcinomas with a median size of 2.5 cm (range, 1–8) in a histopathological study in explanted livers.

The major complication rate in our study was 12.3%, slightly but not significantly higher than the 8.8% rate in the control group with random non-subdiaphragmatic tumors. The majority of complications such as pleural effusion or pneumothoraces were relatively easy to treat. Significant bleeding in all but one instance could be immediately managed with angiographic coiling in the same general anesthesia session. Rhim et al [29] reported a low complication rate of 4% (1/25) with artificial ascites during RF ablation of subdiaphragmatic tumors, and Kim et al [5] described no major complications in a small series of 15 patients treated by RF ablation with an angulated transhepatic approach. More recently, Vo Chieu et al [18] reported major complication rates of up to 57.9% with microwave ablation in subcapsular hepatic dome tumors with a transpleural approach while Ding et al [20] reported major complication rate of 10% (6/60) with a transhepatic approach. Asvadi et al [22] showed that creation of artificial ascites is a valuable option for avoiding diaphragm-associated complications. Application of this technique would have most likely prevented most of the complications related to thermal injury of the diaphragm in our patient series. In unresectable HCCs, Zhang et al (16) reported a major complication rate of 4.4% after combined TACE-RF and Zhao et al (19) of 2.3% after TACE and 9.4% after RF ablation, respectively. The complication rate of our study is moderately higher, which may be due in part to the larger size of our treated tumors and the larger number of inserted needles (median 9 needles, ranging from 3 to 22). Supporting this assumption, a subanalysis of our data showed that the complication rate in larger tumors treated with more RF probes was significantly higher. However, the major complication rate of the present study compares favorably with the reported rates following laparoscopic or open liver resection of primary or metastatic liver tumors in the posterosuperior segments of the liver. According to the in-surgical studies that primarily used Clavien-Dindo classification [30], the major complicate rate in the present study was 4.9% (10/204). Major complication rates after laparoscopic RFA were 10% and 12.4%, respectively [20, 31]. Several surgical studies report significantly higher major complicate rates of 10–27% after LLR, and 18–37% after open resection, respectively [10, 32–35].

Overall survival rates after SRFA of colorectal liver metastases [16], breast cancer liver metastases [36], intrahepatic cholangiocellular carcinomas [37], and melanoma liver metastases [38] are comparable with those of liver resection. In the current study, patients with exclusively hepatic dome HCCs showed OS rates at 1, 3, and 5 years from the date of the first SRFA of 87.1%, 76.2%, and 58.2% and 91%, 75.8%, and 55.2% for matched controls. The median OS rates were 66.9 months and 72 months, respectively. Ding et al [20] treated 60 patients with HCC with percutaneous RFA and 56 patients with laparoscopic RFA. The OS rates for the percutaneous RF ablation group were 91.7%, 56.7%, and 36.7 at 1, 3, and 5 years with a median OS of 44 months. The corresponding rates for the laparoscopic RF ablation group were 89.2%, 57.1%, and 44.6 at 1, 3, and 5 years with a median of 57 months, respectively. Kim et al [39] reported high 3- and 5-year OS rates of 84% and 72.7% in HCC patients with 12% of tumors abutting the diaphragm with a mean tumor size of 2.1 cm. Llovet et al [40] showed in a RCT the survival benefit of TACE vs. a control group with significantly higher 1- and 2-year OS of 82% and 63% vs. 63% and 27%, respectively. Especially in larger tumors, several studies reported evidence for the benefit of a combination therapy of TACE and RF ablation; Zhang et al [26] reported 1-, 2-, and 3-year OS rates of 89%, 61%, and 43% after combined RF ablation and TACE in patients with HCC beyond the Milan criteria.

The DFS for patients with exclusively hepatic dome HCC was 78%, 47.5%, and 35.6% at 1, 3, and 5 years and for matched controls 67.2%, 50.8%, and 35.5%, respectively. These data compare with Kim et al [39], who reported similar recurrence-free survival rates after RF ablation of 66.5%, 20.4%, and 17% at 1, 3, and 5 years.

### Limitations

Study limitations include the retrospective design, heterogeneity of treatments (adjunctive TACE or chemotherapy), and single-center treatment bias. Comparisons with previous related studies are limited as stereotactic navigation systems were not employed in prior reports.

In conclusion, stereotactic RFA is a feasible, safe, and efficacious option in the management of difficult-to-treat hepatic tumors abutting the diaphragm.

## Abbreviations

DFS: Disease-free survival
HCC: Hepatocellular carcinoma
ICC: Intrahepatic cholangiocarcinoma
IN: Incomplete necrosis
LR: Local recurrence
OS: Overall survival
RF: Radiofrequency
SRFA: Stereotactic radiofrequency ablation
TACE: Transcatheter arterial chemoembolization
